# Gout and rheumatoid arthritis are associated with subclinical vascular damage, reduced brachial vasoreactivity and coronary microvascular dysfunction: a case-control study

**DOI:** 10.1007/s00296-025-05868-6

**Published:** 2025-04-23

**Authors:** Oğuz Konal, Furkan Bölen, Tolga Sinan Güvenç, Özlem Pehlivan, Sevilay Batıbay, Fatma Betül Özcan, Ayşe Paralı, Feyza Aksu, Şeref Kul, Mustafa Çalışkan

**Affiliations:** 1 Department of Cardiology, Istanbul Medeniyet University, Dumlupınar D100 Karayolu No:98, Kadıkoy, Istanbul, 34720 Turkey; 2 Department of Cardiology, Faculty of Medicine, Istinye University, Istanbul, Turkey; 3 Department of Rheumatology, Istanbul Medeniyet University, Dumlupınar D100 Karayolu No:98, Kadıkoy, Istanbul, 34720 Turkey

**Keywords:** Gout, Rheumatoid arthritis, Atherosclerosis, Vascular damage

## Abstract

Gout and rheumatoid arthritis (RA) are distinct disorders, but both of these inflammatory arthropaties are associated with a relatively similar risk of atherosclerotic cardiovascular disease. We aimed to assess carotid intima-media thickness (CIMT), flow-mediated vasodilatation (FMD) and coronary flow reserve (CFR) in both disorders to understand and compare the extent of vascular damage. Patients with recently diagnosed gout (*n* = 30) and RA (*n* = 40) without previous cardiovascular disease were included to the present study, and 45 healthy volunteers served as controls. All participants underwent ultrasonographic examinations to measure CIMT, FMD and CFR. In unadjusted analysis, both gout and RA were associated with a significantly higher CIMT and significantly lower CFR as compared to controls, while FMD was only lower in the gout group. After adjustment, there was a significantly higher odds ratio (OR) for CIMT (OR:7.02, 95%CI:2.45–20.58 for gout and OR:2.86, 95%CI:1.27–6.57 for RA) and a lower OR for CFR (OR:0.21, 95%CI:0.08–0.55 for gout and OR:0.17, 95%CI:0.07–0.45 for RA) in both groups, while only gout patients had a lower OR for FMD (OR:0.21, 95%CI:0.08–0.55). There were no significant differences or evidence of a difference between gout and RA patients for CIMT, FMD or CFR. Gout and RA are associated with a similar degree of vascular damage, which may explain the similar incidence of atherosclerotic cardiovascular disease in both diseases.

**Graphical Abstract:**

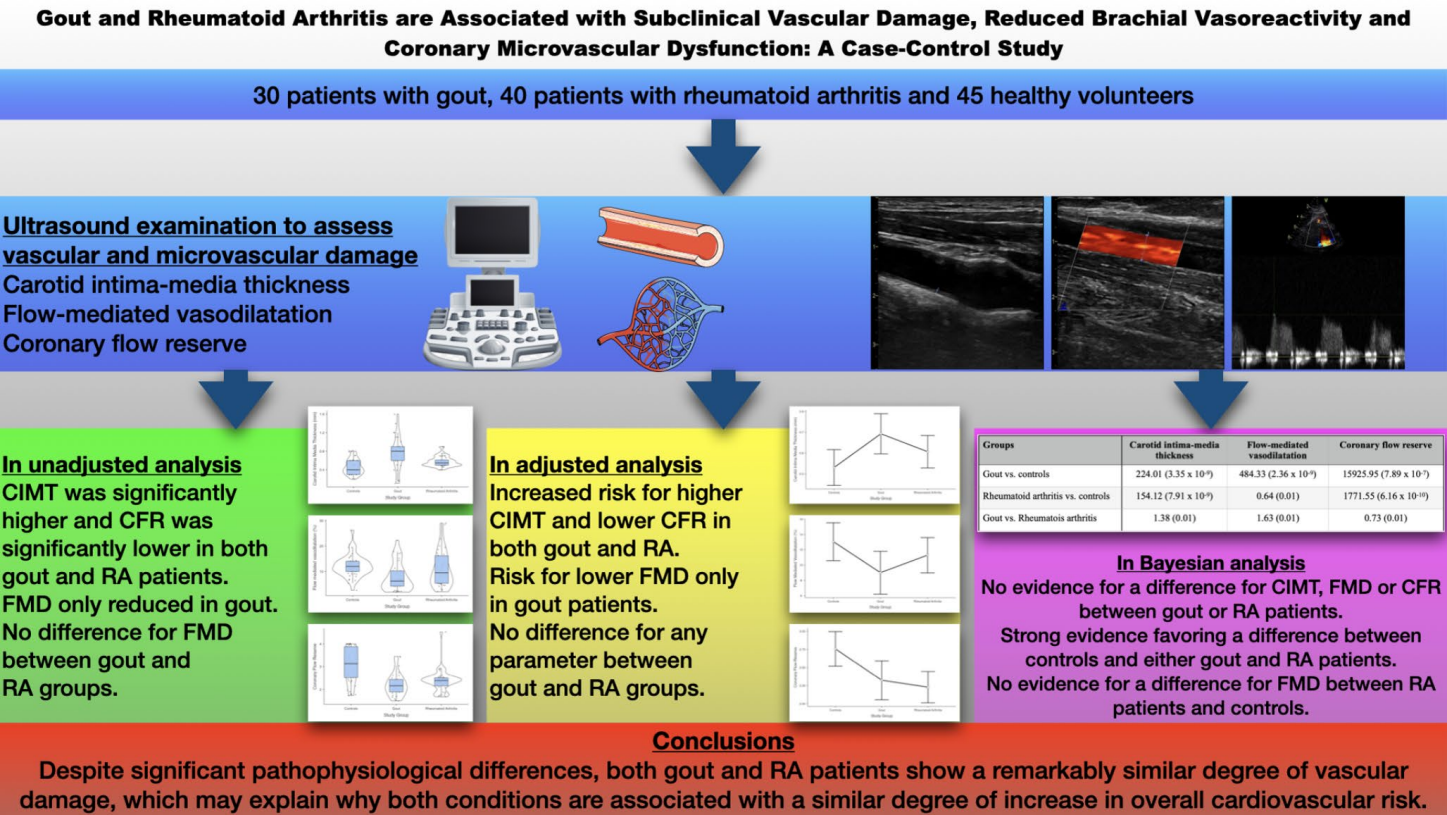

**Supplementary Information:**

The online version contains supplementary material available at 10.1007/s00296-025-05868-6.

## Introduction

Gout is an inflammatory arthropathic disorder that is caused by the accumulation of monosodium urate crystals within the synovium [1]. Hyperuricemia and inflammation, two major features of gouty arthritis, are linked to accelerated atherosclerosis and an increased risk of cardiovascular disorders [2–4]. Not surprisingly, several studies have also shown an association between gout and various cardiovascular disorders, especially those related to atherosclerosis [4, 5]. In a large case-control study, the relative risk increase for cardiovascular disease was 49% in men and 88% in women, respectively [5].

As gout is associated with multiple pathophysiologic mechanisms linked to atherosclerotic cardiovascular disease (ASCVD), it remains unclear whether the ASCVD risk attributable to gout is similar to that of other inflammatory arthropathies. As suggested in animal models, hyperuricemia may promote inflammation and induce endothelial dysfunction [2, 6]. Accumulation of uric acid in atherosclerotic lesions and tophi formation were suggested as other possible pathways explaining the association of hyperuricemia with ASCVD [7–9]. Finally, hyperuricemia is a marker for the overproduction of free radicals, which are also associated with ASCVD [4].

Rheumatoid arthritis (RA) is the archetypal inflammatory arthropathy that is characterized by autoimmune activation within the synovial membrane [10]. Although RA is associated with a significant increase in the risk of ASCVD, this increased risk is in large part mediated through inflammation without the hyperuricemia or associated crystal formation that is characteristic of gout [11–13]. Thus, a comparison of the vasculopathic effects of gout and RA could provide valuable insights into the basic pathophysiological mechanisms leading to increased ASCVD risk in both disorders.

In the present study, we aimed to understand the effects of gout and RA on the indices of peripheral and coronary vascular injury, including flow-mediated vasodilatation (FMD), coronary flow reserve (CFR) and carotid intima-media thickness (CIMT), particularly after adjustment for confounders. Additional Bayesian analyses were performed to assess the strength of evidence for the associations between gout, RA and vascular damage.

## Methods

This study was performed as part of an ongoing institutional registry that enrolls patients with various cardiac and non-cardiac diseases. Per registry protocol, all subjects undergo a thorough vascular assessment that includes ultrasonographic measurement of FMD, CFR and CIMT. For the present analysis, subjects ≥ 18 years of age with a recent diagnosis of gout or RA were included. Those with an acute gout flare, known established ASCVD or atherosclerotic peripheral vascular disease, significant valvular disease, cardiomyopathy of any type, persistent arrhythmias, additional systemic disease (including malignancy or infections) at the time of enrollment, grade 3 or more chronic kidney disease or significant liver disease, chronic lung disease or pulmonary hypertension were excluded. Out of 86 patients that were initially screened, 30 patients with gout and 40 patients with RA were included to this study. 45 random volunteers that fulfilled the above mentioned exclusion criteria were included to the study to serve as controls. The design of the study and workflow was summarized in Supplementary Fig. 1.

Participants’ demographic and clinical data were collected via direct interviews or using the institutional electronic medical database. A screening examination, basic transthoracic echocardiography and an exercise stress test with imaging for those deemed at high risk for ASCVD were done prior to enrollment. All ultrasonic examinations were done by a single investigator experienced in measurement of CFR, FMD and CIMT.

The study was conducted in accordance with 1964 Declaration of Helsinki and its subsequent revisions. Patients gave their written informed consent prior to inclusion, and the study was approved by a local ethics committee (Medeniyet University Clinical Research Ethics Committee, Decision Date: 24.05.2023, Document No: 2023/0334).

### Diagnosis of gout and rheumatoid arthritis

A diagnosis of RA and gout were made by a rheumatology specialist according to the American College of Rheumatology / European League Against Rheumatism criteria for RA (2010 criteria) and gout (2015 criteria) [14, 15]. All patients were included to this present study soon after a diagnosis is established, before the initiation of any medical treatment for either condition.

### Biochemical analyses

Samples were collected after overnight fasting, and all collected samples were transferred to the institutional laboratory within 1 h of collection. Biochemical studies, including C-reactive protein and uric acid, were done according to the standardized protocols. Complete blood counts were obtained using a Coulter autoanalyzer.

### Carotid intima-media thickness

All subjects were placed in a comfortable supine position and CIMT was measured after resting for 15 min at room temperature. A 12.5 mHz linear probe connected to a Vivid S6 (GE Vingmed Ultrasound, Horten, Norway) echocardiography device was used for CIMT measurement. A 1 cm segment within the first 2 cm distal region from the common carotid artery bulb was determined and measurements were performed from this region. Following the determination of the right and left CIMT, the mean CIMT was calculated for each participant by averaging these measurements.

### Flow-mediated dilatation

Flow-mediated dilatation (FMD) was assessed by ultrasonographic measurement of the brachial artery in the dominant arm. Patients were asked to refrain from excessive exercise half an hour before the procedure. They were also advised to avoid nicotine-containing foods and other vasoactive substances that may affect endothelial function. Patients were placed in the supine position and the brachial artery was palpated in the longitudinal plane in the antecubital fossa. Measurements were performed with a Vivid S6 (GE Vingmed Ultrasound, Horten, Norway) echocardiography machine using a 12.5 MHz linear probe. The brachial artery was imaged longitudinally in the region of best visualization along the tracing, and a segment between the lumen and vessel wall with clear discrimination of the anterior and posterior intimal surfaces was selected for two-dimensional imaging. Brachial artery diameter was measured three times at the end of diastole according to ECG monitoring and the average of these three measurements was taken and this value was recorded as the baseline brachial artery diameter. After basal values were recorded, the cuff pressure was increased to 200 mmHg or 50 mmHg above the patient’s systolic blood pressure for complete cessation of arterial flow and ischemia was induced by keeping the cuff in this position for 5 min. Images of the brachial artery were obtained 60 s after the cuff was lowered. The mean of three different measurements was used to record the post-flow brachial artery lumen diameter. FMD was expressed as % (percent) increase relative to baseline vessel diameter.

### Measurement of coronary flow reserve

Coronary flow reserve of all patients included in the study was measured with a Vivid S6 GE echocardiography device using a 7.5 MHz transducer in the lateral decubitus state. Adenosine was used as the hyperemic agent for transthoracic measurement of CFR. The mid-distal portion of the left anterior descending artery in the interventricular septum was visualized in the modified two-chamber window. Coronary flow velocities were recorded at baseline and after intravenous administration of adenosine at 140 mg/kg/min for 4 min. The highest 3 measurements in each recording were averaged. Coronary flow reserve was defined as the ratio of maximum diastolic flow velocity during vasodilatation to basal diastolic flow velocity at rest.

**Fig. 1:**
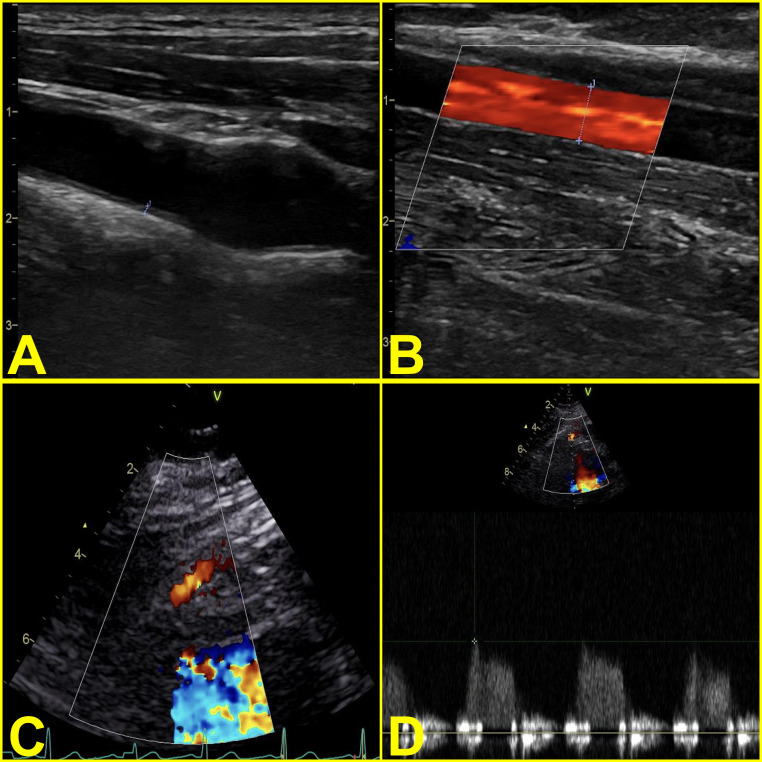
Examples showing the measurement of carotid intima-media thickness (**A**), flow-mediated vasodilatation in brachial artery following cuff release (**B**), visualization of left anterior descending artery in the modified apical view (**C**) and measurement of peak diastolic velocity (**D**)

Examples for measurements were provided in Fig. 1.

### Statistical analysis

Continuous variables were given as mean ± SD if the pattern of distribution was normal or as median and interquartile range if the pattern of distribution was skewed, while percentages were given for categorical variables. The patterns of distribution and homogeneity of variances assumptions were analyzed using Shapiro-Wilk and Levene tests, respectively. For continuous parameters with a normal distribution, unadjusted comparisons between groups were done with one-way ANOVA test with Welch correction when necessary and Tukey’s HSD or Games-Howell tests were used as post-hoc analyses. For parameters with a skewed distribution, Kruskal-Wallis test was used and Dwass-Stein-Critchlow-Fligner test was used for post-hoc analyses. For categorical parameters, unadjusted comparisons were done using chi-squared test or Fisher test as appropriate, and Bonferroni correction was used when comparisons were necessary between two groups. Ordinal logistic regression models were built for univariable and multivariable analyses to determine the associations between study groups with CIMT, FMD and CFR. Multivariable models were adjusted for possible confounders (age, gender, body-mass index, hypertension, diabetes, smoking and total cholesterol), and all parameters were included in the model without using a selection criterion. For between-group analyses, Bonferroni-corrected p values were given. Finally, a Bayesian ANCOVA model that adjusted for the same covariates was built to understand the strength of evidence supporting the associations between markers of vascular damage, gout and RA. For this latter analysis, square-root and logarithmic transformations were applied to CIMT, FMD and CFR as necessary.

Three sensitivity analyses, excluding patients with hypertension, smoking and diabetes in each analysis, were performed to understand the relative contribution of these conditions on the main results. Kruskal-Wallis test were done to determine differences between subgroups and post-hoc analyses were done using Dwass-Stein-Critchlow-Fligner test.

Recorded images for 30 randomly selected cases (10 cases from each group) were re-analyzed by the same investigator initially made the measurements and by a second investigator, and intraclass correlation coefficients for average measures were calculated to determine interobserver and intraobserver measurement variabilities for CIMT, FMD and CFR, as well as for the other echocardiographic measurements that were reported in the study.

For all frequentist analyses, the level of significance was set at 0.05. For Bayesian analyses, Bayesian factors (BF_M_ and BF_10_) were provided to judge to strength of evidence. Bayesian factors between 3 and 10, 10–30 and > 30 were accepted as signifying moderate, strong and very strong evidence favoring the alternative hypothesis, respectively. All statistical analyses were done using Jamovi (The jamovi project (2024). jamovi. (Version 2.5) for MacOs. Retrieved from https://www.jamovi.org.) and SPSS 27.1 for MacOs (IBM Inc, Armonk, NY) softwares.

## Results

Mean age of the overall sample was 45.4 ± 12.2 years, and 75 participants (65.2%) were male. 30 patients (26.1%) had gout, while 40 patients (34.8%) had RA and 45 subjects (39.1%) served as controls. Median rheumatoid factor (U/ml), anti-citrullinated protein antibody (U/ml), Disease Activity Score with C-Reactive Protein and Simple Disease Activity Index for RA patients were 74.8 (29.5–150.0), 28.8 (11.6–54.0), 2.04 (1.80–2.73) and 1.50 (1.28–4.67), respectively. All subjects had CIMT and CFR measurements, while FMD measurements were absent in 2 patients with RA.

Table 1 summarizes demographic, clinical, laboratory and echocardiographic features in patients with gout or RA. On overall, gout patients were older, were more likely to be male, smoking, had more comorbidities such as obesity or hypertension and had higher triglycerides. As expected, both gout and RA patients had higher C-reactive protein (CRP) concentrations as compared to controls, while gout patients had significantly higher uric acid concentrations compared to the two other groups.

**Table 1:** Demographic, clinical and echocardiographic characteristics of the study groups and controls. P values that were below 0.05 were given in bold. LA, left atrium; LDL, low-density lipoprotein; LV, left ventricle

Characteristic	Gout (*n* = 30)	Rheumatoid Arthritis (*n* = 40)	Controls (*n* = 45)	*P* value
**Demographic and clinical characteristics**				
Age (years)	54.0 ± 10.2^a, c^	44.7 ± 8.8	40.4 ± 10.3	**< 0.001**
Sex (%Female)	5 (16.7%)^c^	22 (55.0%)^b^	13 (28.9%)	**0.002**
Body mass index (kg/m^2^)	29.6 ± 3.4^a, c^	25.8 ± 2.7	26.5 ± 3.7	**< 0.001**
Hypertension (%)	13 (43.3%)^a, c^	8 (20.0%)	6 (13.3%)	**0.009**
Diabetes (%)	3 (10.0%)	7 (17.5%)	3 (6.7%)	0.34
Smoking (%)	14 (46.7%)^a,^	14 (35.0%)	8 (17.8%)	**0.02**
Median symptom duration^*^ (mo)	3.5 (2.0–5.0)	7.0 (3.9–13.0)	-	**0.003**
**Laboratory findings**				
Hemoglobin (g/dl)	14.8 ± 1.2^c^	13.4 ± 1.5^b^	14.4 ± 1.5	**< 0.001**
Leucocyte count (x10^3^/mm^3^)	7.2 ± 1.5	8.5 ± 3.3^b^	6.9 ± 1.5	**0.03**
C-reactive protein^*^ (mg/dl)	3.31 (1.00–6.47)^a^	3.86 (2.14–6.12)^b^	1.60 (0.84–2.00)	**< 0.001**
Total cholesterol (mg/dl)	190.0 ± 46.2	182.0 ± 37.2	181.0 ± 39.8	0.22
LDL-cholesterol (mg/dl)	113.0 ± 28.5	109.0 ± 31.9	98.7 ± 37.6	0.15
Triglycerides^*^ (mg/dl)	234.0 (202.0–312.0)^a, c^	97.5 (80.6–137.0)^b^	162.0 (81.0–189.0)	**< 0.001**
Creatinine (mg/dl)	0.93 ± 0.19^a, c^	0.82 ± 0.28	0.80 ± 0.14	**0.004**
Uric acid (mg/dl)	6.47 ± 1.60^a, c^	4.13 ± 1.68^b^	5.00 ± 1.30	**< 0.001**
**Echocardiographic variables**				
Indexed LV end-diastolic volume (ml/m2)	52.0 ± 8.7	51.8 ± 9.8	51.5 ± 8.7	0.97
Indexed LV end-systolic volume (ml/m2)	15.6 ± 3.9	16.3 ± 4.3	16.2 ± 4.1	0.69
LV ejection fraction (%)	70.0 ± 6.3	68.4 ± 6.7	68.3 ± 6.8	0.36
Indexed LV mass (g/m^2^)	80.3 ± 14.3^a^	87.8 ± 14.1^b^	69.4 ± 12.7	**< 0.001**
LA diameter (mm)	35.3 ± 3.5^c^	31.9 ± 3.4^b^	34.0 ± 4.5	**< 0.001**

* This variable had a skewed distribution pattern

^a^*p* < 0.05 for the comparison between gout patients vs. controls

^b^*p* < 0.05 for the comparison between patients with rheumatoid arthritis vs. controls

^c^*p* < 0.05 for the comparison between gout patients vs. patients with rheumatoid arthritis

**Fig. 2:**
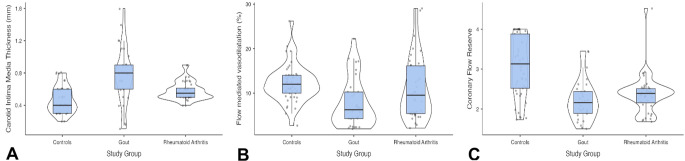
Violin plots showing unadjusted values for carotid intima-media thickness (**A**), flow-mediated vasodilatation (**B**) and coronary flow reserve (**C**) measurements in controls, gout patients and patients with rheumatoid arthritis. Points show individual patient data

**Fig. 3:**
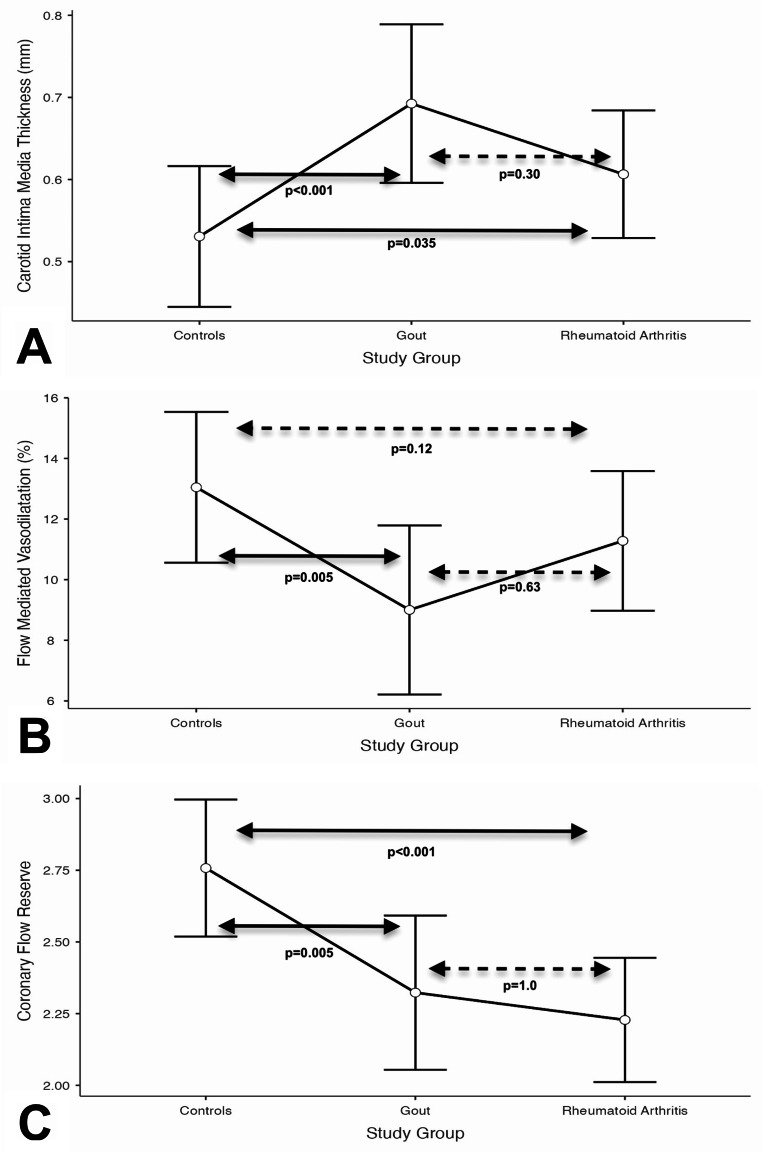
Adjusted mean and standard deviation values for carotid intima-media thickness (**A**), flow-mediated vasodilatation (**B**) and coronary flow reserve (**C**) values in controls and study groups. Solid arrows show post-hoc comparisons that were statistically significant, while interrupted arrows show non-significant comparisons

Findings for the markers of vascular damage were presented in Table 2. Both gout and RA patients had significantly higher CIMT and significantly lower CFR as compared to controls, while there were no differences between the two study groups for either parameter. In contrast, FMD was only lower in gout patients (as compared to controls), but there were no significant differences between gout and RA groups for this parameter (Fig. 2). Similar findings were obtained in the logistic regression models after adjustment for possible confounders, with patients in both groups having significantly higher CIMT and significantly lower CFR than controls after adjustment, while only gout patients had a significantly lower FMD as compared to controls (Table 3). For all three parameters of vascular damage, there were no significant differences between gout and RA groups after adjustment (*p* > 0.05 for all) (Fig. 3).

**Table 2:** Measurements for the vascular damage and microvascular function in study groups and controls. P values that were below 0.05 were given in bold

Characteristic	Gout (*n* = 30)	Rheumatoid Arthritis (*n* = 40)	Controls (*n* = 45)	*P* value
Carotid intima-media thickness^*^ (mm)	0.80 (0.60–0.90)^a, c^	0.55 (0.50–0.61)^b^	0.40 (0.30–0.60)	**< 0.001**
Flow-mediated vasodilatation^*^ (%)	6.28 (4.28–10.3)^a^	9.53 (5.41–16.1)	10.0 (12.0–14.0)	**0.001**
Coronary flow reserve^*^ (%)	2.16 (1.89–2.44)^a^	2.39 (2.15–2.51)^b^	3.13 (2.52–3.88)	**< 0.001**

* This variable had a skewed distribution pattern

^a^*p* < 0.05 for the comparison between gout patients vs. controls

^b^*p* < 0.05 for the comparison between patients with rheumatoid arthritis vs. controls

^c^*p* < 0.05 for the comparison between gout patients vs. patients with rheumatoid arthritis

**Table 3:** Univariable and multivariable ordinal logistic regression analysis for the associations between carotid intimal-media thickness, flow-mediated vasodilatation and coronary flow reserve with study groups. Multivariable analyses were adjusted for age, gender, body mass index, hypertension, diabetes, smoking status and total cholesterol. CI, confidence interval; OR, odds ratio

Group	Univariable Analysis	Multivariable analysis
	OR (95%CI)	OR (95%CI)
	Carotid Intima-Media Thickness	
**Gout (vs. controls)**	17.28 (6.43–48.80)	7.02 (2.45–20.58)
**Rheumatoid arthritis (vs. controls)**	3.45 (1.62–7.51)	2.86 (1.27–6.57)
**Rheumatoid arthritis (vs. gout)**	0.20 (0.08–0.50)	0.41 (0.14–1.15)
	**Flow Mediated Vasodilatation**	
**Gout (vs. controls)**	0.18 (0.08–0.43)	0.21 (0.08–0.55)
**Rheumatoid arthritis (vs. controls)**	0.52 (0.24–1.13)	0.41 (0.17–0.96)
**Rheumatoid arthritis (vs. gout)**	2.87 (1.17–7.18)	1.94 (0.69–5.53)
	**Coronary Flow Reserve**	
**Gout (vs. controls)**	0.08 (0.03–0.19)	0.21 (0.08–0.55)
**Rheumatoid arthritis (vs. controls)**	0.15 (0.06–0.34)	0.17 (0.07–0.41)
**Rheumatoid arthritis (vs. gout)**	1.98 (0.87–4.54)	0.84 (0.32–2.20)

On Bayesian analyses, there were very strong evidence to support a difference between the three groups for CFR (BF_M_: 1738) and FMD (BF_M_: 123.8), while there was only moderate evidence to support a difference for CIMT (BF_M_: 9.6). Unadjusted Bayesian factors for each individual group were provided in the Table 4. To note, there was no evidence supporting a difference between gout and RA patients for all of the markers of vascular damage (BF_10_ < 3 for all).

**Table 4:** Bayesian analyses showing bayesian factors for the associations between carotid intimal-media thickness, flow-mediated vasodilatation and coronary flow reserve with study groups. Numbers in parentheses show standard errors. BF_10_, bayesian factor for the alternative hypothesis

Groups	Carotid intima-media thickness	Flow-mediated vasodilatation	Coronary flow reserve
	BF_10_ (error%)	BF_10_ (error%)	BF_10_ (error%)
Gout vs. controls	224.01 (3.35 × 10^− 9^)	484.33 (2.36 × 10^− 9^)	15925.95 (7.89 × 10^− 7^)
Rheumatoid arthritis vs. controls	154.12 (7.91 × 10^− 9^)	0.64 (0.01)	1771.55 (6.16 × 10^–10^)
Gout vs. Rheumatois arthritis	1.38 (0.01)	1.63 (0.01)	0.73 (0.01)

Results for the sensitivity analyses were given in Supplementary Table 1. All major findings for the differences between groups regarding to CIMT, FMD and CFR were replicated in the three sensitivity analyses, apart from the lack of difference between groups for FMD after excluding smokers. To note, approximately half of the patients with gout and one-thirds of patients with RA were excluded from this latter analysis.

For CIMT, FMD and CFR; intraclass correlation coefficients for intraobserver variabilities ranged from 0.924 to 0.953, while intraclass correlation coefficients for interobserver variabilities was between 0.864 and 0.899. For the remaining echocardiographic variables that were reported, intraobserver variabilities ranged between 0.938 and 0.70 and interobserver variabilities ranged in between 0.829 and 0.936.

## Discussion

Although inflammatory arthropathies are characterized with an increase in adverse cardiovascular events and mortality, relatively little is known about the underlying mechanisms. Main takeaways from the present study are as follows: (i) compared with healthy volunteers, both gout and RA are associated with vascular damage, (ii) these associations remained significant after adjustment for confounders related to atherosclerosis, particularly those that are commonly observed in gout patients, (iii) there were no significant differences between RA and gout in terms of coronary microvascular dysfunction or subclinical carotid atherosclerosis, iii) although brachial vasoreactivity appeared to be more impaired in gout patients as compared with RA in the unadjusted analyses, this appears to be a function of confounding factors in the gout patients rather than being a true difference. See the graphical abstract for a summary of the study design and key findings.

Although gout and RA are distinct diseases from a pathophysiologic standpoint, with gout being a “deposition disease” and RA being characterized by autoimmune activation, both disorders share significant similarities in terms of the patterns of immune activation. Both disorders are result in the formation of neutrophil extracellular traps (NETs), which causethe extrusion of DNA and other intracellular components from dead neutrophils to create traps that catch and eliminate pathogenic agents [16]. These NETs could damage endothelial lining and induce endothelial dysfunction (ED), linking these pathogenic processes to atherosclerosis [17, 18]. Moreover, monosodium urate crystals that accumulate in gout induce the formation of the NLRP3 inflammasome, which is activated early in vascular inflammation [19, 20]. There is also some evidence for activation of NLRP3 inflammasome pathway in RA [21]. Apart from immune mechanisms, both gout and RA are associated with abundant production of small reactive compounds called reactive oxygen species (ROS) [22, 23]. Formation of ROS within the bloodstream and endothelial lining leads to modification and oxidation of lipoproteins, endothelial dysfunction and vascular damage [24].

Therefore, the basic mechanisms of vascular damage appear to be similar in these two “inflammatory arthropathies”, and as predicted by this statement, present results suggest that the degree of vascular damage did not differ between gout and RA. Although both CIMT and FMD were numerically worse in the gout group, present findings suggest that this difference may in part reflect the presence of demographic factors (i.e. older age, male predominance) and comorbid conditions (i.e. obesity, smoking) that favor atherogenesis in the gout group as we found no evidence for a difference in the adjusted analyses. Also, the extent of reduction in CFR was similar in both groups, suggesting a similar degree of involvement of the coronary microvasculature. Although both CIMT and FMD have been associated with cardiovascular events in epidemiologic studies, both reflect vascular damage in peripheral arteries rather than coronary arteries. In contrast, CFR is a direct indicator of ASCVD and coronary microvascular dysfunction and a strong predictor of both fatal and non-fatal cardiovascular events [25-27]. As such, from a pathophysiological perspective the present results may help to explain the strikingly similar increase in cardiovascular events in gout and RA [28].

It has long been debated whether the increased incidence of ASCVD events in gout is a direct result of the underlying disease processes or is related to demographic factors and comorbid conditions associated with atherosclerosis [28-30]. Present findings establish gout itself as a determinant of vascular damage, but also suggest that the some degree of vascular damage can be explained by the comorbid conditions. This is was particularly true for CIMT and CFR, where more than half of the variance was associated with conditions common in gout patients, such as older age, higher BMI or smoking. Also, the association between gout and reduced FMD was not observed after excluding smoking patients in the sensitivity analysis, although this may simply reflect the reduced statistical power of this latter analysis given that approximately half of the patients within the gout group were smokers. As some of these risk factors are correctable, it is reasonable to assume that adverse cardiovascular outcomes in gout patients could in principle be reduced by risk factor interventions. Whether the treatment of gout is associated with a reduction in cardiovascular events is less clear. Colchicine, a microtubule inhibitor that is used to treat gout flares, is associated with a reduction in cardiovascular events in the overall population in two large RCTs [31, 32]. A meta-analysis of observational studies that included gout patients did not suggest a reduction in cardiovascular events such as myocardial infarction, although there was a significant reduction in mortality [33]. There is some observational data suggesting an improvement in endothelial function after lowering uric acid, but the clinical translation of these findings is uncertain since no RCTs to date have found a reduction in cardiovascular events with treatments aimed to lower uric acid [34-37]. Although the association between gout and cardiovascular events appears to be causal, as also suggested by this present work, there is still inadequate evidence to prove that treating the underlying abnormalities in gout (i.e. inflammation or hyperuricemia) would correct or at least stall underlying vascular damage.

In contrast, several lines evidence suggest that disease-modifying anti-rheumatic drugs (DMARDs) are associated with a reduced risk for ASCVD [38-40]. Interestingly, this reduction in ASCVD risk occurs despite the dyslipidemic effects of DMARDs and benefits of these drugs (such as methotrexate) do not appear to extend beyond patients with RA [41, 42]. There is some data to suggest that beneficial effects of DMARDs may be mediated via a reduction in vascular damage and improvement in microvascular function [43, 44]. Thus, the available evidence for a causal relationship between inflammation, RA and ASCVD is more convincing, whereas similar evidence is insufficient for other inflammatory arthropathies.

The present study has several advantages and limitations. Our study group has extensive experience in performing coronary flow measurements and other markers of vascular damage within an acceptable variability, which increases the reliability of the present results [43-47]. However, we report results from a single center and the sample size was relatively small, with a limited power to detect significant differences between groups. Although there was little evidence of a true difference between the gout and RA groups in Bayesian analyses, which allow evaluation of the strength of evidence to be assessed without the need for a significance test, it cannot be ruled out that the results could have been somewhat different if the sample size had been larger. As patients were recruited soon after diagnosis, present results are not applicable to patients who were on treatment or had long-lasting disease. While patients were not on urate-lowering drugs or anti-inflammatory agents (including steroids, colchicine and DMARDs) at the time of enrollment, the use of over-the-counter non-steroidal anti-inflammatory drugs by patients themselves prior to enrollment could not be excluded. Since the majority of patients with gout or RA had a risk factor for cardiovascular disease, confounding effects of these conditions could not be completely ruled out even after statistical adjustment. However, excluding these conditions would not only limit the number of patients eligible for the study, but also reduce the generalizability of the results, as most patients with gout and RA in the population have these risk factors. Although adjustments were made for detectable confounders, no causal inferences could be drawn because adjustments could not be made for all observed and unobserved confounders. Finally, adjustments could be made only for a limited number of confounders to maintain the robustness of the regression analyses.

## Conclusions

Present findings suggest a similar degree of vascular damage in gout and RA, which may help explain the similarities between these two inflammatory arthropathies in terms of cardiovascular outcomes. To some extent, vascular damage is related to demographic and clinical factors, particularly in patients with gout. However, even in patients with gout the degree of vascular damage could not be explained by confounding factors alone, thus suggesting a residual risk attributable to the disease processes themselves. Whether treatment of the diseases themselves could prevent or reverse vascular damage remains uncertain, particularly for gout, emphasizing the need for further clinical and translational studies.

## Electronic supplementary material

Below is the link to the electronic supplementary material.

Supplementary Material 1

Supplementary Material 2

## Declarations

### Open data sharing

The data that support the findings of this study are available from the corresponding author (MC), upon reasonable request.

### Editing support and use of AI

No editorial support from professional agencies or AI support was used for this study.
