# Supplementary material for: Gout and rheumatoid arthritis are associated with subclinical vascular damage, reduced brachial vasoreactivity and coronary microvascular dysfunction: a case-control study
Source: Rheumatol Int. 2025 Apr 23;45(5):117. doi: 10.1007/s00296-025-05868-6 (PMC12018609; doi:10.1007/s00296-025-05868-6)
Supplement: Supplementary file 1 — Supplementary Material 1 [file 296_2025_5868_MOESM1_ESM.docx]

| Characteristic | Gout | Rheumatoid Arthritis | Controls | P value |
| --- | --- | --- | --- | --- |
| **Excluding patients with hypertension** | | | | |
| Carotid intima-media thickness^*^ (mm) | 0.80 (0.60 - 0.90)^a,c^ | 0.58 (0.50 - 0.63)^b^ | 0.40 (0.30 - 0.60) | **<0.001** |
| Flow-mediated vasodilatation^*^ (%) | 7.32 (4.44 - 10.3)^a^ | 8.98 (5.37 - 13.8) | 12.0 (10.2 - 14.0) | **0.006** |
| Coronary flow reserve^*^ (%) | 2.22 (1.97 - 2.43)^a^ | 2.39 (2.26 - 2.51)^b^ | 3.22 (2.59 - 3.95) | **<0.001** |
| **Excluding patients with smoking** | | | | |
| Carotid intima-media thickness^*^ (mm) | 0.80 (0.55 - 0.90)^a^ | 0.58 (0.50 - 0.60)^b^ | 0.40 (0.30 - 0.50) | **<0.001** |
| Flow-mediated vasodilatation^*^ (%) | 9.53 (4.60 - 12.3) | 8.33 (5.41 - 13.2) | 12.0 (10.0 - 14.0) | 0.10 |
| Coronary flow reserve^*^ (%) | 2.12 (1.93 - 2.44)^a^ | 2.33 (2.13 - 2.50)^b^ | 3.26 (2.75 - 3.96) | **<0.001** |
| **Excluding patients with diabetes** | | | | |
| Carotid intima-media thickness^*^ (mm) | 0.80 (0.60 - 0.95)^a,c^ | 0.52 (0.50 - 0.60)^b^ | 0.40 (0.30 - 0.60) | **<0.001** |
| Flow-mediated vasodilatation^*^ (%) | 5.71 (3.41 - 9.97)^a^ | 9.68 (5.33 - 16.3) | 11.3 (9.48 - 14.00) | **<0.001** |
| Coronary flow reserve^*^ (%) | 2.17 (1.94 - 2.39)^a^ | 2.39 (2.21 - 2.50)^b^ | 3.14 (2.55 - 3.92) | **<0.001** |

**Supplementary Table 1.** Sensitivity analysis for the associations between study groups and indices of vascular damage and microvascular injury. P values that were below 0.05 were given in bold.

* This variable had a skewed distribution pattern.

^a^ p<0.05 for the comparison between gout patients vs. controls

^b^ p<0.05 for the comparison between patients with rheumatoid arthritis vs. controls

^c^ p<0.05 for the comparison between gout patients vs. patients with rheumatoid arthritis
