# Supplementary figures and images for: Gout and rheumatoid arthritis are associated with subclinical vascular damage, reduced brachial vasoreactivity and coronary microvascular dysfunction: a case-control study
Source: Rheumatol Int. 2025 Apr 23;45(5):117. doi: 10.1007/s00296-025-05868-6 (PMC12018609; doi:10.1007/s00296-025-05868-6)

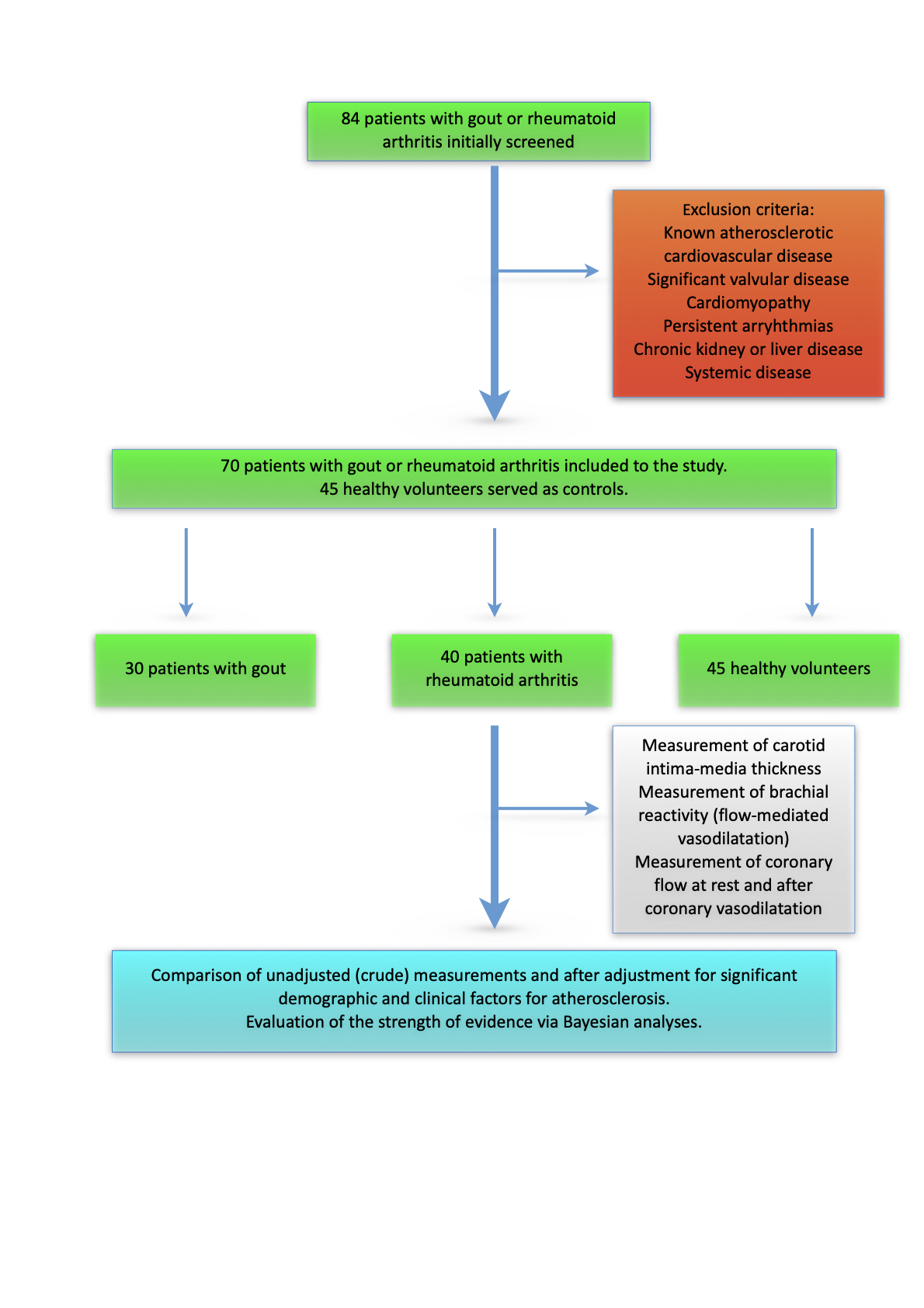

Supplement: Supplementary file 2 — Supplementary Material 2 [file 296_2025_5868_MOESM2_ESM.jpeg]
